# Systemic Treatment for Hepatocellular Carcinoma Recurrence After Liver Transplantation

**DOI:** 10.3390/curroncol33030141

**Published:** 2026-02-28

**Authors:** Chiara Mazzarelli, Francesco Berardi, Alessandra Bonfichi, Marina Clemente, Michele Orlando, Marina Strollo, Luca Saverio Belli

**Affiliations:** 1 Hepatology & Gastroenterology Unit, ASST GOM Niguarda, 20162 Milan, Italy; francesco.berardi@ospedaleniguarda.it (F.B.); alessandra.bonfichi1@universitadipavia.it (A.B.); marina.clemente@ospedaleniguarda.it (M.C.); michele.orlando@ospedaleniguarda.it (M.O.); marina.strollo@ospedaleniguarda.it (M.S.); luca.belli@ospedaleniguarda.it (L.S.B.); 2 Department of Biomedical Sciences, Humanitas University, Pieve Emanuele, 20072 Milan, Italy; 3 Department of Internal Medicine, IRCCS San Matteo Hospital Foundation, University of Pavia, 27100 Pavia, Italy; 4 Department of Medicine and Surgery, University of Milano-Bicocca, 20900 Monza, Italy

**Keywords:** hepatocellular carcinoma, liver transplantation, systemic treatment

## Abstract

Hepatocellular carcinoma (HCC) is a leading cause of cancer-related mortality worldwide, and liver transplantation (LT) remains the only curative treatment addressing both tumor burden and underlying liver disease. Despite an adequate candidate selection, HCC recurrence after LT occurs in 8–20% of cases and is associated with a poor prognosis, particularly in patients who experience an early relapse. The management of HCC recurrence remains particularly challenging due to the lifelong immunosuppression required after LT, which may promote tumor progression and restrict therapeutic options. This review synthesizes the current evidence on systemic therapies for recurrent HCC after LT, focusing on tyrosine kinase inhibitors (TKIs), immunotherapy, and the current available immunosuppression strategies. Unfortunately, in this setting, robust prospective studies are lacking, and clinical decision-making remains based on retrospective data and expert consensus. Future research should prioritize the prospective evaluation of systemic regimens, integration of immunosuppression modulation, and careful exploration of immunotherapy or new target therapies in this special population.

## Simple Summary

Hepatocellular carcinoma (HCC) is a leading cause of cancer-related mortality worldwide. Liver transplantation (LT) represents the only curative treatment for both HCC and the underlying liver disease; however, post-transplant recurrence occurs in 8–20% of recipients and is associated with a poor prognosis. The management of recurrent HCC after LT is particularly challenging due to the need for lifelong immunosuppression, which may promote tumor progression and limit therapeutic options. In patients with advanced or multifocal recurrence, systemic therapy is the mainstay of treatment. Optimization of immunosuppressive regimens, particularly calcineurin inhibitor minimization and selective use of mTOR inhibitors, is critical.

## 1. Introduction

Hepatocellular carcinoma (HCC) is a major contributor to cancer-related morbidity and mortality worldwide [1], being the sixth most common diagnosis of cancer and the third most common cause of death in 2020 [2].

Liver transplantation (LT) remains the only therapeutic approach with curative potential for both HCC and the underlying liver disease [3]. It should be considered not only in patients with early-stage HCC but also in appropriately selected patients with multifocal or locally advanced tumors after a successful downstaging [3,4]. The implementation of stringent selection criteria, such as the Milan criteria [3], and subsequent expanded criteria [5,6], has substantially improved post-transplant survival rates. However, despite the careful selection of candidates, HCC recurrence post-LT remains a challenge, as it occurs in approximately 8–20% of recipients and is associated with limited treatment options and a poor prognosis, with a median survival of 7 to 16 months [7,8,9]. Most recurrences are diagnosed during the first 2–3 years. Early recurrence (<12 months) is a strong predictor of poor outcomes [10,11], while late recurrence is associated with better survival and is more amenable to curative intent treatment with superior survival outcomes [12,13,14,15]. HCC recurrence has been associated with several oncological risk factors, such as tumor burden at LT, histological aggressiveness, vascular invasion and alpha-fetoprotein levels [9,15], as well as several non-oncological risk factors, including immunosuppressive drug exposure [16] or CMV reactivation after LT [17]. The management of HCC recurrence is extremely challenging as the post-transplant setting is characterized by the need to maintain a lifelong immunosuppression to prevent graft rejection. However, immunosuppressive drugs can influence tumor biology, impact on drug–drug interactions, and limit the efficacy and safety of potential treatment options, from surgery to systemic agents [8,18,19,20]. Immunosuppression withdrawal has been theorized to lower the risk of oncologic relapse, constituting a potentially significant benefit in the management of post-transplant recurrence [21,22,23,24].

**Figure 1:**
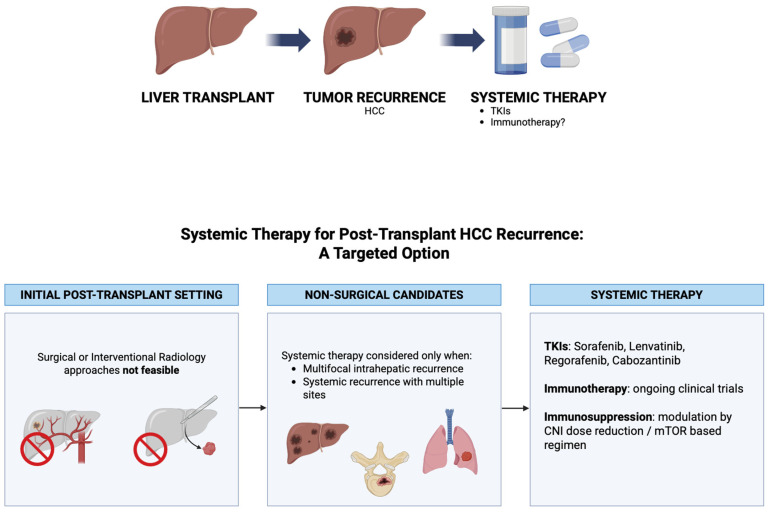
Hepatocellular carcinoma recurrence after liver transplantation: treatment possibilities according to disease recurrence.

A tailored approach is recommended, where therapeutic decisions are made according to disease and patient-specific variables, including the timing and pattern of recurrence, patient outcomes, graft function, and biological markers [25]. A multidisciplinary team (MDT) including transplant hepatologists, surgeons, oncologists, and radiologists is important to maintain the complex balance between oncological efficacy and the preservation of the liver allograft. Patients with limited, oligometastatic recurrence, a good performance status (ECOG 0–1), and preserved liver graft function (Child–Pugh A, ALBI grade 1) are suitable candidates for treatment, primarily surgical resection [11,26,27]. In patients with recurrent HCC following liver transplantation, those having well-differentiated tumors and an absence of microvascular involvement have the most favorable outcomes following surgical resection, with one-year survival rates greater than 70% [28,29]. Patients unsuitable for surgery may be considered for locoregional therapies, although these approaches are less effective in the presence of poorly differentiated tumors and microvascular involvement [25,29]. However, patients with advanced, multifocal or multi-metastatic disease, with a poorer performance status (ECOG ≥ 2), or impaired graft function (Child–Pugh B and/or clinically significant portal hypertension) should be candidates for systemic treatments or palliative care [21,30] (Figure 1).

The advent of tyrosine kinase inhibitors (TKIs), such as Sorafenib (SOF) and Lenvatinib (LEN), provided the first effective systemic options for advanced HCC [22,23]. Consequently, TKIs have also been applied in the post-transplant setting [20,23,24], mostly at a personalized dose due to the comorbidities and concomitant medications of LT patients. More recently, the scenario of advanced HCC treatment has been completely changed by the advent of immune checkpoint inhibitor (ICI)-based regimens, like Atezolizumab/Bevacizumab (A/B) or Durvalumab/Tremelimumab (DT). ICI-based treatments are superior to TKIs in terms of overall survival (OS) and progression-free survival (PFS) [31,32]. Consequently, these treatments have become the first-line therapy in non-transplant populations with unresectable or advanced HCC [30,33]. However, their application in transplant recipients is challenging due to the risk of inducing allograft rejection and consequently data on the use of ICIs in transplanted patients are scarce [21,34,35]. Historically, the LT population has been excluded from clinical trials evaluating novel cancer therapies, leading to a paucity of high-level evidence to guide clinical decision-making [22,23,34].

This literature review aims to comprehensively evaluate the current evidence for systemic treatment strategies for HCC recurrence after LT. We will critically examine the efficacy and safety of TKIs, discuss the challenges and limited role of immunotherapy, the role of immunosuppression in the management of patients with HCC recurrence and explore emerging therapeutic considerations and future directions for optimizing outcomes in this special population. A comprehensive understanding of systemic therapeutic strategies in the context of sustained chronic immunosuppression is pivotal for optimizing clinical outcomes in patients with HCC recurrence after LT.

## 2. Methods

A literature review was conducted by searching the PubMed database using the keywords “hepatocellular carcinoma”, “HCC recurrence”, “liver transplantation” and “systemic therapy”. Studies published in English that addressed recurrence of HCC after liver transplantation were included. Reference lists of eligible articles were also screened to identify additional relevant studies. Relevant information was extracted from selected studies, including study design, patient characteristics, immunosuppressive regimens, and the main oncological outcomes following liver transplantation.

## 3. TKI Treatment

Tyrosine kinase inhibitors have been the gold-standard for advanced HCC since 2007 [22]. TKIs act by binding to the active site of tyrosine kinases, preventing the phosphorylation of target proteins and subsequently downgrading signal transduction. In HCC, the blockade of key tyrosine kinase pathways—particularly those involving vascular endothelial growth factor receptors (VEGFRs), epidermal growth factor receptors (EGFR/ERBB), and platelet-derived growth factor receptors (PDGFRs)—can effectively suppress tumor growth and angiogenesis. The primary molecular targets of TKIs in HCC include VEGFRs, PDGFRs, and other receptor tyrosine kinases involved in tumor vascularization and proliferation. TKIs exert their effects through either reversible or irreversible binding to various domains of receptor tyrosine kinases (RTKs), thereby inhibiting receptor activation. This inhibition disrupts downstream signaling cascades, including the MAPK, PI3K/AKT/mTOR, and JAK/STAT pathways, resulting in reduced tumor cell growth, proliferation, and metastasis [36]. An overview of the major tyrosine kinase inhibitors (TKIs) investigated in HCC, along with their efficacy in general and immunosuppressed patients, is summarized in Table 1.

Despite immunotherapy having largely supplanted TKIs in the treatment of HCC, TKIs remain particularly relevant for transplant recipients due to the risk of liver graft rejection associated with immunotherapy [35]. This aspect will be discussed in more detail below. Prospective ongoing studies about the use of TKI agents for the treatment of HCC recurrence are reported in Table 2.

### 3.1. Sorafenib

Systemic treatments for the recurrence of HCC after LT are based on therapies targeting multiple tyrosine kinases [21]. Among them, SOF is a multi-kinase inhibitor that interferes with tumor cell proliferation and angiogenesis by blocking RAF kinases, vascular endothelial growth factor receptors (VEGFRs), and platelet-derived growth factor receptors (PDGFRs). SOF was the first agent to demonstrate a survival benefit in patients with advanced or unresectable HCC. In two Phase III randomized trials, SOF improved median OS by approximately three months compared to placebo (OS 10.7 vs. 7.9 months, *p* < 0.001 in the SHARP trial [22] vs. OS 6.5 vs. 4.2 months, *p* = 0.014 in the Asia-Pacific trial) [44]. However, LT recipients were excluded from these pivotal registration trials; therefore, evidence in this population is largely derived from retrospective observational studies. Since 2009, several case series have assessed the safety and efficacy of SOF in LT recipients with HCC recurrence, both as monotherapy and in combination with mTOR inhibitors (mTOR-Is), given their dual immunosuppressive and antitumor properties [45]. Sposito et al. have demonstrated that SOF improves the OS of patients with HCC recurrence after LT who were not suitable for surgery or radiotherapy (10.6 vs. 2.2 months) compared to best supportive care (BSC) [40]. Similar findings were also reported in an Asian population cohort. Kang et al. have confirmed that SOF significantly improved median post-transplant survival in patients with HCC recurrence (14.2 vs. 6.8 months) compared to BSC, with an acceptable toxicity profile [46]. A similar study reported that patients treated with SOF for HCC recurrence achieved a disease control rate of 73.4%, with a one-year OS rate of 60% [45]. The efficacy of SOF has been further corroborated by a large multicenter retrospective study involving 81 post-transplant patients, which reported a median OS of approximately 19 months following initiation of TKI therapy [46]. The improved prognosis reported in the most recent publications has been attributed to a superior performance status, the application of multimodal or sequential treatment strategies and a better management of TKI-related adverse events [47]. High-risk pathological features such as poor differentiation and vascular invasion predict recurrence risk after LT [48]. However, no studies have demonstrated that specific histological subtypes significantly influence Sorafenib efficacy in the post-LT recurrence context. Vascular invasion is a well-established prognostic risk factor of HCC recurrence after LT. However, evidence supporting its role as a predictive factor for response to Sorafenib in the setting of post-transplant recurrence remains limited. In the general population, macrovascular invasion is associated with poorer survival and may correlate with reduced Sorafenib efficacy, reflecting a more aggressive tumor biology [49]. However, Sposito et al. demonstrated that vascular invasion was not independently associated with survival after recurrence, and the benefit of Sorafenib was maintained regardless of vascular invasion [40].

mTOR-Is, commonly used as immunosuppressive agents, target complementary signaling pathways implicated in HCC progression, providing synergistic antitumor and immunosuppressive effects. Consequently, several studies have investigated combination therapies with mTOR-Is and TKIs to enhance treatment efficacy. A retrospective study by Gomez-Martin et al. [50] reported that the combination of SOF and mTOR-Is appears to be more effective than SOF alone in terms of OS (21.3 vs. 11.8 months). The authors reported minor and manageable adverse events (grade 1–2), although two serious bleeding complications were reported, highlighting the need for a careful risk assessment of bleeding risk in this population. The safety of early SOF administration in LT recipients has been evaluated in an Italian cohort including 50 patients. SOF-related adverse events (AEs) occurred in all treated patients, 56% of them being grade 3–4. There was no significant difference in SOF-related AEs between patients taking mTOR-Is or other immunosuppressive regimens, except for hand–foot syndrome [51]. In this cohort, the use of mTOR-Is and SOF was associated with an increase in OS in univariate analysis but not in multivariate analysis. Thus, SOF represents the most extensively studied systemic therapy for HCC recurrence after LT, demonstrating a survival benefit over BSC in retrospective studies, both as monotherapy and in combination with mTOR-Is. However, considering several adverse effects related to SOF treatment, a careful individualized assessment is required.

### 3.2. Regorafenib

Regorafenib (REG) is an oral multi-kinase inhibitor used in the treatment of various types of oncological diseases, including colorectal cancer, gastrointestinal stromal tumors and hepatocellular carcinoma (HCC). REG is an oral diphenyl urea multi-kinase inhibitor that targets angiogenic (VEGFR1-3, TIE2), stromal (PDGFR-β, FGFR), and oncogenic receptor tyrosine kinases (KIT, RET, and RAF). In the setting of HCC, REG proved to be particularly effective in increasing OS in patients with progressive disease after SOF with a good toxicity profile [39]. Similar efficacy has been demonstrated in LT recipients with HCC recurrence after LT. In a multicenter observational retrospective study, Iavarone et al. reported no life-threatening or unexpected adverse events attributable to REG administration in an LT population. In this study, the majority of AEs were classified as mild or moderate (Grade 1 or 2 according to CTCAE grading), and notably, no cases of hepatic graft rejection were recorded. REG was associated with a median OS of approximately 12.9 months. When considering sequential therapy with SOF followed by REG, the median OS extended to 38.4 months [52]. These results have been further confirmed in 2021 in a larger multicenter study. Patients receiving REG as a second-line treatment after SOF exhibited a median OS of approximately 28.8 months, compared to a median OS of 15.3 months in patients who did not receive REG [53]. REG was generally well tolerated in LT recipients, with adverse events being manageable. The combination of mTOR-Is with REG was not associated with any significant impact on OS in LT patients with HCC recurrence [53]. In a more recent multicenter analysis, Ozbay et al. reported that the median OS for patients treated sequentially with SOF followed by REG was 35.9 months, demonstrating an improvement in OS compared to the previous studies [42]. In conclusion, REG appears to positively impact OS with an acceptable safety profile and can be considered a valid second-line treatment option for patients with HCC recurrence after LT who have progressed on SOF.

### 3.3. Cabozantinib

Cabozantinib (CABO) is a multi-tyrosine kinase inhibitor (TKI) targeting VEGFR-1, 2, and 3, MET, and AXL, and is approved as a second-line systemic therapy for patients with advanced HCC who have progressed on SOF based on the CELESTIAL trial, which demonstrated improved OS (10.2 vs. 8 months; *p* = 0.004) and PFS (5.2 vs. 1.9 months; *p* < 0.0001) compared to placebo [38,54]. However, its application in LT setting is less defined, primarily because transplant recipients were excluded from pivotal registration trials. Consequently, data regarding CABO efficacy and safety in this specific population are lacking and the few data available are mainly derived from case reports or small series [24,55,56]. Invernizzi et al. previously reported that CABO was safe and effective as a second- or third-line treatment in patients with HCC recurrence after LT. Among the 10 patients who received CABO as a second-line therapy, the median OS from CABO initiation was 15.6 months (95% CI: 10.1–21.0), and 37.6 months (95% CI: 21.6–53.6) from the initiation of the first-line therapy. No liver rejection was observed, and no drug-to-drug interactions with immunosuppressive drugs were reported in this cohort. The combination treatment with CABO and mTOR-Is was not explored in this study [41]. Further data about the use of CABO in post-LT setting are awaited from a prospective trial. Azhie et al. have designed and initiated a prospective, single-arm, non-comparative, open-label Phase II clinical trial (NCT04204850) to specifically evaluate CABO (60 mg once daily) in patients with recurrent HCC after LT [57]. This study utilizes an optimal two-stage design, planning to enroll a total of 17 patients, with the primary endpoint being the disease control rate (DCR) at 4 months. Secondary objectives include PFS, OS, and a detailed assessment of safety and tolerability, alongside correlative biomarker analyses. The results of this ongoing trial are expected to yield critical prospective data regarding the efficacy and safety of CABO in the post-LT setting. In conclusion, CABO appears to be a feasible and potentially effective therapeutic alternative for recurrent HCC after LT, although robust prospective data is still needed to better define its role in this specific population.

### 3.4. Lenvatinib

Lenvatinib (LEN) is an oral multi-kinase inhibitor (MKI) that targets vascular endothelial growth factor (VEGF) receptors 1–3, fibroblast growth factor receptors 1–4, platelet-derived growth factor receptor alpha (PDGFRα), RET and KIT. Notably, prospective trials evaluating LEN in HCC have excluded patients with a history of LT, resulting in a paucity of data regarding its use in this specific clinical context [23]. A retrospective analysis involving 56 patients with recurrent HCC following liver transplantation compared outcomes between those treated with LEN (*n* = 14) and SOF (*n* = 42). The LEN group demonstrated a significantly longer median OS of 15.0 months (95% CI: 11.5–31.5), compared to 7.8 months (95% CI: 4.0–15.4) in the Sorafenib cohort (*p* = 0.017). After adjustment for potential confounding variables, including alpha-fetoprotein levels and recurrence patterns, LEN treatment remained independently associated with improved survival outcomes [42]. Comparable findings were also reported by Magyar et al., who demonstrated a 2.3-fold improvement in OS from the initiation of LEN therapy compared to SOF [58]. A recent multicenter retrospective analysis by Bang et al. reported a median progression-free survival (PFS) of 7.6 months (95% CI: 5.3–9.8) and a median OS of 14.5 months (95% CI: 0.8–28.2) with LEN treatment [43]. The safety profile observed in this post-transplant population was consistent with findings from the REFLECT trial and real-world studies in non-transplanted patients [37]. Importantly, patients with preserved liver function (ALBI grade 1) at treatment initiation experienced significantly better survival outcomes compared to patients with graft dysfunction. In the few published cohorts, immunosuppressive agents did not appear to negatively influence the efficacy of LEN in the treatment of HCC after LT or to increase adverse events compared to non-transplanted patients [23,43,58]. The impact of histological features and vascular invasion patterns on LEN response has not yet been explored. Thus, LEN may represent a valid first-line systemic treatment option for recurrent HCC after LT. Notably, LEN should be favored over SOF as an initial systemic therapy in patients with preserved graft function and stable immunosuppression, given its advantages related to OS and PFS, in addition to its more favorable safety profile. Indeed, LEN may be particularly advantageous in patients who have previously developed Sorafenib-related dermatological toxicities, notably hand–foot skin reactions.

## 4. Immunotherapy

The liver is recognized as a tolerogenic organ, a property that is essentially related to its constant exposure to a vast array of antigens derived from the gut microbiota. This immunotolerant environment facilitates immune homeostasis and prevents excessive inflammatory responses [59,60,61]. However, effective immunosurveillance remains critical, particularly in the context of malignant transformation, where immune activation is necessary to eradicate emerging tumor cells. The rationale for immunotherapy in HCC is fundamentally based on this dual role of hepatic immune regulation [21]. Programmed death-1 (PD-1/PD-L1) inhibitors primarily activate CD8+ T cells against tumor cells, while cytotoxic T-lymphocyte antigen 4 (CTLA4) inhibitors decrease the number of CD4+ T reg cells and consequently reduce the immunosuppressive environment [62].

Since 2020, immunotherapy has become the first treatment option for unresectable HCC. In fact, it has been demonstrated that combined treatments with A/B or D/T are superior in terms of OS and PFS compared to SOF [31,32]. However, in HCC recurrence after LT, the role of ICIs remains undefined. Only a limited number of studies have included patients receiving concurrent immunosuppressive therapy, primarily due to concerns over the potential risk of life-threatening graft rejection. As a result, TKIs currently represent the preferred therapeutic option in this population [34,35]. Consequently, data on the efficacy of ICIs after LT remain limited, primarily based on case reports and small published retrospective series [63,64,65]. The most comprehensive review published, involving 39 patients treated with ICIs post-LT for recurrent HCC, reported an acute graft rejection rate of 20.5% with a mortality rate of 50% among those cases [63]. Moreover, in the same cohort, the authors observed that ICIs are less effective compared to in an immunocompetent population. In fact, only 25.6% of the population showed a disease control rate, while 46.2% experienced a disease progression. However, these data are limited and must be carefully considered, considering that a large percentage of patients prematurely stopped the treatment due to clinical rejection.

Patients receiving PD-1/PD-L1 inhibitors may have a higher incidence of liver graft rejection compared to those treated with CTLA-4 inhibitors [34,35,63,64]. The frequency of immune-mediated rejection appears to be higher in non-hepatic solid organ transplant recipients, possibly due to the intrinsic tolerogenic nature of the liver [60]. The use of combination immunosuppressive regimens has also been associated with a lower risk of immune-related graft injury [63]. Immunosuppressive regimens based on calcineurin inhibitors were associated with a lower rejection rate (20%) compared to those based on mTOR inhibitors (80%) [63]. Nonetheless, susceptibility to rejection may vary based on factors such as the timing post-transplant, prior episodes of progressive rejection, and individual immune profiles [61,66].

Limiting the high risk of graft rejection associated with immunotherapy in patients with advanced recurrent HCC after liver transplantation requires mandatory patient stratification. Optimal candidates may include patients with a longer post-transplant interval, no high-risk genetic variants—such as the CTLA4 +49A/+6230G haplotype 1—and low or absent PD-L1 expression in the graft. Positive PD-L1 expression in HCC tissue has been associated with a significantly higher incidence of graft rejection (66.7%) compared to zero cases observed among patients with negative PD-L1 expression [67]. In patients with PD-L1-negative allografts, a more tolerogenic liver microenvironment may be present, potentially mediated by immune checkpoints and co-inhibitory pathways involving CD160; the B- and T-lymphocyte attenuator (BTLA); herpesvirus entry mediator (HVEM); and LIGHT, a ligand for the lymphotoxin-β receptor [67,68]. Assessment of donor-specific antibodies and a liver biopsy should be considered to exclude the presence of subclinical rejection before starting ICI therapy. In addition, the characterization of a potentially high-risk PD-L1-positive graft is also relevant. According to Shi et al., a graft is considered PD-L1-positive when PD-L1-expressing cell, including hepatocytes, lymphocytes, and Kupffer cells, represent ≥1% of the total number of viable cells, assessed by immunohistochemistry [67]. Furthermore, to mitigate the risk of graft rejection in liver transplant recipients undergoing ICIs, prophylactic administration of corticosteroids—such as prednisolone—has also been proposed prior to the initiation of immunotherapy [69]. In a recent meta-analysis, the combined use of steroids and mTOR inhibitors in patients exposed to immunotherapy was associated with a lower risk of rejection compared with other immunosuppressive agents [70]. Routine monitoring of liver function tests is strongly recommended in liver transplant recipients receiving ICIs, to permit an early detection of immune-mediated liver injury [64].

While combined therapy with ICIs and TKIs is the mainstay in the management of HCC in non-transplanted patients, this combination therapy is unexplored in the post-liver transplant setting. The majority of published reports have described the use of single-agent immunotherapy, primarily PD-1 or PD-L1 inhibitors, with only a limited number of case reports evaluating combination approaches [71,72]. The use of immunotherapy-based combinations has mainly been reported as a salvage strategy. For example, a favorable outcome was described in a 35-year-old male treated with Atezolizumab and Bevacizumab for recurrent fibrolamellar hepatocellular carcinoma, having a 10-month survival benefit [72]. Furthermore, a 57-year-old male with recurrent HCC after LT demonstrated a radiological response to Pembrolizumab combined with Sorafenib following disease progression on Sorafenib monotherapy [71]. In addition, Di Marco et al. reported a small cohort of five liver transplant recipients treated with a combination of Nivolumab and Bevacizumab as a second-line therapy following progression on TKI treatment [73]. The authors demonstrated that this regimen was feasible in the post-transplant setting, with a limited and manageable toxicity profile. Notably, the risk of allograft rejection appeared to be moderate, although the small sample size suggests this may not be conclusive.

In conclusion, although ICI-based regimens are superior to TKI-related OS and PFS as a first-line treatment for unresectable HCC in the general population, their superiority has not yet been demonstrated in the liver transplant population. In addition, the risk of graft rejection remains high. Thus, current guidelines do not recommend the use of an ICI-based regimen in the treatment of HCC recurrence after LT unless in a clinical trial [30]. Prospective studies on ICIs for the treatment of HCC recurrence are currently ongoing and the main studies are reported in Table 3.

## 5. Combined Treatment Strategies

Combining systemic therapy with other therapeutic approaches could be an option in the HCC recurrence after LT setting. While data are still scarce in the literature, emerging evidence suggests that integrating systemic therapy with locoregional treatments in unresectable HCC recurrence may offer superior survival benefits compared to monotherapy. In a study that included 106 patients with HCC-R post-LT, 52 were treated with a combination of TACE plus SOF, while 54 were treated with TACE alone [74]. The combination group revealed a better median overall survival (17 vs. 10 months) and progression-free survival (12 vs. 6 months) in comparison with the TACE-only group, without an increase in adverse events. Another retrospective study including 70 patients treated with TACE in combination with either SOF or LEN, followed by Regorafenib administration, showed a median post-recurrence survival of 16 months, a median post-transplant survival of 25 months and an objective response rate (ORR) of up to 71%, with adverse events reported as manageable [75].

Another possible combination strategy consists of the use of systemic agents as an adjuvant therapy after LT for HCC. Several studies have been conducted with SOF as an adjuvant treatment, initially with promising results in small cohorts, especially in patients beyond Milan criteria [76,77]. However, more recently available data show no substantial benefit to its use as a preemptive treatment in LT recipients [78]. Regarding LEN, preliminary retrospective evidence suggests that its use as an adjuvant treatment may delay recurrence and lower recurrence risk after LT, particularly in high-risk pathological subgroups, but definitive conclusions are limited by study design and cohort size [79,80]. Consequently, larger and more robust studies are required to definitively establish the efficacy of this agent in this clinical setting.

## 6. Immunosuppression Management

Calcineurin inhibitors (CNIs) remain the cornerstone of immunosuppressive therapy in patients with LT, with tacrolimus (Tac) generally selected over cyclosporin due to superior graft survival outcomes [81,82]. Experimental models have demonstrated that Tac exerts a dose-dependent pro-oncogenic effect, mediated through upregulation of transforming growth factor-β1 (TGF-β1) [83].

**Figure 2:**
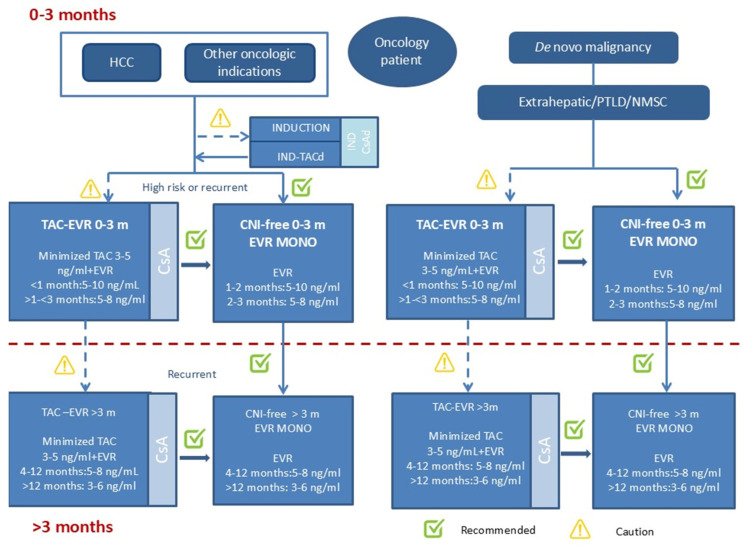
Immunosuppressive management in oncology patients with LT proposed by the Italian Liver Transplant working group.

Clinically, two large cohort studies reported that LT recipients who developed de novo malignancies exhibited higher Tac trough levels. It has been demonstrated that colorectal, pulmonary, and skin cancers are related to Tac dose overexposure [84,85]. In patients transplanted for HCC, early Tac troughs > 10 ng/mL are linked to a two-fold increase in recurrence risk of HCC after LT [86]. Therefore, current guidelines suggest an early minimization of Tac, with target levels of 6–10 ng/mL during the first month and around 4 ng/mL in the long term. mTOR-I inhibitors, such as sirolimus and everolimus (EVR), have a certain relevance, given their antiproliferative effects and established role in several cancers [87]. In fact, mTOR-Is are approved for the treatment of several malignancies including neuroendocrine tumor, advanced rectal colon cancer, astrocytoma, pancreatic cancer and certain breast cancers. Because of their recognized anti-neoplastic effects, mTOR-Is have been extensively studied in the prevention of malignancies common in transplant recipients, specifically skin cancers and HCC [88]. Retrospective studies and meta-analyses suggest sirolimus may reduce HCC recurrence by up to 50%, although these findings are limited by selection bias [89,90]. The Silver Trial, a large, randomized trial, found no overall benefit of sirolimus but did demonstrate improved outcomes in low-risk patients within Milan criteria at five years [91]. An analysis of the US Scientific Registry of Transplant Recipients also reported favorable, though not statistically significant, outcomes in terms of lower HCC recurrence and cancer-specific mortality among recipients treated with sirolimus-based immunosuppression. Interestingly, these effects appeared to be influenced by the recipient’s age at transplantation, with better outcomes observed in patients older than 55 years compared with those aged ≤ 55 years [90]. Evidence for EVR, the only mTOR inhibitor approved in LT, is still emerging, with ongoing trials evaluating its role in patients beyond Milan criteria. Preliminary data from a Phase II trial have demonstrated that the combination of EVR + a reduced dose of Tac vs. a standard Tac dose is associated with similar efficacy and safety, and with an improvement in renal filtrate, and it showed a possible reduction in HCC recurrence beyond Milan criteria at 24 months, though a longer follow-up is needed [92]. A recent nationwide study from Korea found that adding EVR to CNIs significantly reduces the risk of HCC and extrahepatic cancers in LT recipients [93]. However, robust evidence from prospective clinical trials is currently lacking, and the available data do not support the routine use of mTOR inhibitors as monotherapy. Nevertheless, given the need to minimize CNI exposure to reduce nephrotoxicity and potential oncogenic effects, combination regimens based on reduced-dose CNIs and mTOR-Is are recommended—particularly in high-risk patients, such as those with vascular invasion or poor tumor differentiation (Figure 2) [94,95].

Other immunosuppressants, such as anti-metabolites (e.g., mycophenolate), induction agents (e.g., basiliximab), and corticosteroids, influence recurrence only indirectly by enabling CNI minimization. The key challenge in this population is balancing oncologic safety with graft survival. In fact, excessive Tac reduction may increase rejection risk, especially in younger recipients or in those with an underlying autoimmune liver disease [96,97,98,99]. Nowadays, no standardized protocols exist for managing post-LT HCC recurrence, and it remains unclear whether immunosuppression adjustment improves oncologic outcomes. Retrospective studies in de novo malignancies suggest that CNI minimization or withdrawal in favor of mTOR inhibitors may be safe and could possibly confer a certain survival benefit [94,97,99].

## 7. Future Prospectives

Chimeric antigen receptor (CAR) T cell therapy was a major discovery in cancer treatment. Both preclinical and clinical studies have demonstrated the remarkable efficacy of CAR T cells in targeting HCC [100]. CAR T cell therapy combines the cytotoxic power of T cells with the antigen specificity of antibodies by engineering patient-derived T cells to express tumor-specific chimeric antigen receptors. Following in vitro expansion, these modified T cells are reinfused into the patient, where they proliferate and mediate potent, MHC-independent cytotoxicity against tumor cells. Owing to their ability to persist and form memory subsets, CAR T cells act as “living drugs,” capable of inducing durable and highly specific antitumor immune responses [101]. However, the application of CAR T cell therapy in HCC presents unique challenges compared to hematologic malignancies. Several factors contribute to tumor immune resistance such as the lack of tumor-specific antigens in HCC, the presence of underlying liver disease and the highly immunosuppressive tumor microenvironment. It has been demonstrated that the HCC microenvironment is highly immunosuppressive, secondary to different factors such as metabolic restrictions, the dominance of anti-inflammatory cytokines, regulatory immune cells and upregulation of PD-1/PD-L1. In addition, the loss of sinusoidal endothelial fenestrations and the increased extracellular matrix deposition negatively influence CAR T cell infiltration and their cytotoxic activity within the tumor microenvironment. Given the limited efficacy of CAR T cell monotherapy in HCC, combination approaches have been explored to improve outcomes. Some preliminary studies have explored the use of ICIs to restore T cell activity and to amplify CAR T cell cytotoxicity within the tumor environment [102]. Another innovative approach recently proposed is based on the amplification of CAR T cells using mRNA vaccines, which stimulate CAR T cell proliferation and activation directly within the patient, avoiding the need for prolonged ex vivo expansion [103]. CAR T cell therapy is a promising approach for HCC treatment; however, despite the remarkable progress achieved in this field, several challenges remain to be addressed to reduce toxicity, and very limited data comes from the use of CAR T cells in previous transplanted populations. Ongoing prospective studies about the use of innovative treatments are summarized in Table 4.

## 8. Conclusions

The low incidence of HCC recurrence post-LT makes randomized controlled trials difficult to conduct, leading to a paucity of strong clinical data [104]. Notably, current management strategies are largely based on a limited number of retrospective studies with small sample sizes and heterogeneous patient populations, leading to significant uncertainty regarding the optimal therapeutic approach. To date, widely accepted guidelines for the treatment of HCC recurrence after LT are not available, which explains the heterogeneity between transplant centers.

**Figure 3:**
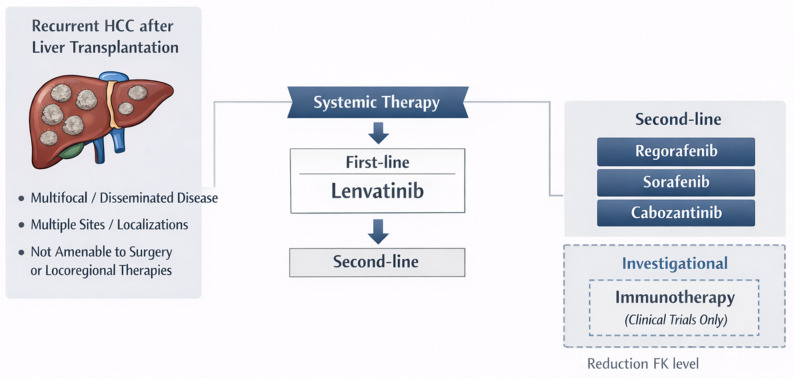
Suggested treatment algorithm for advanced recurrent hepatocellular carcinoma after liver transplantation.

The improvement in liver function following LT allows for the use of multiple therapeutic strategies; however, patient management remains complex due to the need to carefully modulate immunosuppressive therapy and to evaluate potential toxicities and drug–drug interactions. Consequently, careful patient selection, close monitoring, and individualized immunosuppression management are essential. TKI treatment remains the backbone of the treatment of HCC recurrence after LT. Among them, LEN should be preferred over SOF because of its better tolerability, superior efficacy, and lower incidence of adverse events compared with SOF. REG and CABO should be considered as subsequent lines of therapy following progression on LEN. The role of ICIs remains limited in the post-transplant setting due to the high risk of allograft rejection. Nevertheless, further prospective studies are needed to better identify subsets of patients who may safely benefit from ICIs, either as a first-line treatment—such as patients with a long interval from transplantation, viral or metabolic etiologies as the indication for LT, no prior history of rejection, and low PD-1 expression—or as an alternative second-line option after the failure of TKI therapy as a salvage treatment (Figure 3). In conclusion, the management of HCC recurrence after LT remains an unmet clinical need, requiring individualized strategies and prospective studies to define safe and effective treatment algorithms.

## Figures and Tables

**Table 1:** Efficacy outcomes of Sorafenib, Lenvatinib, Regorafenib, and Cabozantinib in advanced hepatocellular carcinoma in non-transplanted and transplanted patients.

DRUG	STUDY	OS (MONTHS)	PFS (MONTHS)	CR (%)	PR (%)	DCR (%)
Sorafenib	Kudo M. (2018) [37]	12.3	3.7	<1	9	60.5
Cabozantinib	Abou-Alfa (2018) [38]	10.2	5.2	0	4	64
Regorafenib	Bruix J. (2017) [39]	10.6	3.1	1	10	65
Lenvatinib	Kudo M. (2018) [37]	13.6	7.4	1	23	75.5
Sorafenib	Sposito C. (2013) [40]	12.9	15.9	0	13.3	73.4
Cabozantinib	Invernizzi F (2021) [41]	15.6	-	-	-	-
Regorafenib	Ozbay MF. (2024) [42]	35.9	5.9	-	51.5	-
Lenvatinib	Bang K. (2023) [43]	14.5	7.6	0	9	88.9

**Table 2:** Prospective trials about use of TKI-based treatment in patients with HCC recurrence after LT.

NCT	Inclusion	Exclusion	Intervention TKI Therapy	Phase	Primary Endpoint	Country	Status
NCT05103904	HCC Recurrence	HIV	Lenvatinib	INT	ORR	USA	Recruiting
NCT04237740	HCC Recurrence	HIV Severe active infection	Relenvatinib	INT	RFS	China	Unknown
NCT04232722	HCC recurrence BCLC B not suitable for local treatment/progression of local treatment and BCLC C	HIV Vascular invasion	Sorafenib combined with Arsenical	INT	ORR	China	Unknown
NCT04204850	Recurrent, measurable HCC post-LT, ECOG 0-1.	Active rejection, brain metastases, active other tumors	Cabozantinib	II	DCR	Canada	Recruiting

Note: The trial status was extracted from ClinicalTrials.gov as per 5 September 2025.

**Table 3:** Current trials about use of ICIs in patients with HCC recurrence after LT.

Title	NCT	Inclusion	Exclusion	Intervention ICI-Therapy	Phase	Primary Endpoint	Country	Status
ImmunoTH	NCT06254248	HCC recurrence (not amendable to LR or surgery) LT > 6 months	ECOG > 2 History of ACR CPT > B	Atezolizumab + Bevacizumab	II	ACR	France	Not yet recruiting
JS001LT	NCT03966209	Recurrent or metastatic HCC after LT not amendable for LR	ECOG > 2 CPT > B	JS001	I	ACR SAER	China	Unknown
	NCT04564313	Pathological confirmed HCC recurrence after LT	CPT > 8 ECOG > 3	Camrelizumab	I	ORR	China	Not yet recruiting

Note: The trial status was extracted from ClinicalTrials.gov as per 5 September 2025.

**Table 4:** Current trials about innovative treatments in patients with HCC recurrence after LT.

NCT	Inclusion	Exclusion	Intervention Therapy	Phase	Primary Endpoi04677088.	Country	Status
NCT04677088	Recurrent locally advanced and/or metastatic HCC post LT HBV + ve	Disease requiring long-term steroids Any other previous cell therapy	TCR-T cells	I	Safety of the TCR-T treatment	China	Unknown
NCT02399735	Pathological confirmed HCC recurrence after LT Same-blood-type relatives without blood-transmitted diseases	Disease requiring long-term steroids	NK cell infusion	INT	GVHD	China	Unknown

## Data Availability

No new data was created or analyzed in this study.

## Abbreviations

The following abbreviations are used in this manuscript:

A/B	Atezolizumab plus Bevacizumab
ACR	Acute Cellular Rejection
AE/AEs	Adverse Event/Adverse Events
AFP	Alpha-fetoprotein
ALBI	Albumin–Bilirubin score
AXL	AXL receptor tyrosine kinase
BCLC	Barcelona Clinic Liver Cancer staging system
BSC	Best Supportive Care
CABO	Cabozantinib
CAR	Chimeric Antigen Receptor
CD4^+^ T reg cells	Cluster of Differentiation 4 regulatory T cells
CD8^+^ T cells	Cluster of Differentiation 8 T cells
CELESTIAL	Phase III randomized trial evaluating Cabozantinib versus placebo in patients with advanced HCC previously treated with Sorafenib
CI	Confidence Interval
CMV	Cytomegalovirus
CNI/CNIs	Calcineurin Inhibitor/Calcineurin Inhibitors
CPT	Child–Pugh–Turcotte score
CTCAE	Common Terminology Criteria for Adverse Events
CTLA-4	Cytotoxic T-Lymphocyte-Associated Protein 4
DCR	Disease Control Rate
DT/D-T	Durvalumab plus Tremelimumab
ECOG	Eastern Cooperative Oncology Group performance status
EVR	Everolimus
FGFR	Fibroblast Growth Factor Receptor
GVHD	Graft-versus-host disease
HCC	Hepatocellular Carcinoma
HBV	Hepatitis B Virus
HIV	Human Immunodeficiency Virus
ICI/ICIs	Immune Checkpoint Inhibitor/Immune Checkpoint Inhibitors
INT	Interventional (study phase)
KIT	KIT proto-oncogene receptor tyrosine kinase
LDLT	Living Donor Liver Transplantation
LEN	Lenvatinib
LR	Locoregional treatment
LT	Liver Transplantation
MET	Mesenchymal–Epithelial Transition factor
MHC	Major Histocompatibility Complex
MKI	Multi-Kinase Inhibitor
MONO	Monotherapy
mRNA	messenger Ribonucleic Acid
mTOR/mTOR-I	mechanistic Target Of Rapamycin/mTOR inhibitors
MVI	Macrovascular Invasion
NCT	National Clinical Trial identifier
NK cells	Natural Killer cells
OBS	Observational (study phase)
ORR	Objective Response Rate
OS	Overall Survival
PD-1	Programmed Death-1
PD-L1	Programmed Death-Ligand 1
PDGFRs	Platelet-Derived Growth Factor Receptors
PET	Positron Emission Tomography
PFS	Progression-Free Survival
RAF	Rapidly Accelerated Fibrosarcoma kinase
REFLECT	Randomized Phase III Study of Lenvatinib vs. Sorafenib in First-Line Treatment of Unresectable HCC
REG	Regorafenib
RET	Rearranged during Transfection proto-oncogene
RFS	Recurrence-Free Survival
SAER	Serious Adverse Event Rate
SHARP	Sorafenib HCC Assessment Randomized Protocol
SOF	Sorafenib
Tac	Tacrolimus
TCR-T	T cell Receptor T cells
TGF-β1	Transforming Growth Factor Beta 1
TIE2	Tyrosine kinase with Immunoglobulin-like and EGF-like domains 2
TKI/TKIs	Tyrosine Kinase Inhibitor / Tyrosine Kinase Inhibitors
UCSF	University of California San Francisco criteria
VEGF	Vascular Endothelial Growth Factor
VEGFRs	Vascular Endothelial Growth Factor Receptors
